# Adverse Event Reporting Quality in Cancer Clinical Trials Evaluating Immune Checkpoint Inhibitor Therapy: A Systematic Review

**DOI:** 10.3389/fimmu.2022.874829

**Published:** 2022-07-07

**Authors:** Yuhong Wang, Chen Chen, Wei Du, Yixin Zhou, Lina He, Shaodong Hong, Li Zhang

**Affiliations:** ^1^ State Key Laboratory of Oncology in South China, Guangzhou, China; ^2^ Collaborative Innovation Center for Cancer Medicine, Guangzhou, China; ^3^ Department of Endoscopy, Sun Yat-Sen University Cancer Center, Guangzhou, China; ^4^ Department of Radiation Oncology, Sun Yat-Sen University Cancer Center, Guangzhou, China; ^5^ Department of Medical Oncology, Sun Yat-Sen University Cancer Center, Guangzhou, China; ^6^ Department of VIP Region, Sun Yat-Sen University Cancer Center, Guangzhou, China

**Keywords:** adverse event, reporting quality, immune therapy, immune checkpoint inhibitor, clinical trials

## Abstract

**Background:**

Immunotherapy has become one of the most important breakthroughs in cancer treatment. Consequently, there have been more immuno-oncology (IO) clinical trials for various cancers in recent decades. However, the quality of such trials in reporting adverse events (AE), especially immune-related AE (irAE), has not been comprehensively evaluated.

**Methods:**

We evaluated the harm reporting quality of IO trials. The PubMed, Embase, Cochrane Library, and Web of Science databases were searched to identify all head-to-head phase II and III clinical trials assessing cancer immunotherapy published between January 1, 2010, and December 31, 2021. Publications were assessed using a 16-point harm reporting quality score (HRQS) derived from the 2004 Consolidated Standards of Reporting Trials (CONSORT) extension. The characteristics associated with improved reporting quality were identified with linear regression.

**Results:**

A total of 123 publications were included. The mean HRQS was 11.1 (range, 5-14). The most common poorly reported items were harms addressed in the title (2%), AE collection methodology (3%), the statistical approach for analyzing harms (11%), and the irAE onset patterns and management (adequately reported in 14% and 33% of publications, respectively). The harm information was well described in the publications’ Results and Discussion sections (89-99%). The multivariable regression model revealed that higher impact factor (IF) (30<IF<60 vs. IF<30, P=0.021) and phase III clinical trial (phase III vs. phase II, P=0.023) were independent predictors of higher quality score.

**Conclusion:**

Our findings show that AE reporting in IO randomized trials is suboptimal. Efforts should be made to improve harm reporting and to standardize reporting practices. Improvements in AE reporting would permit more balanced assessment of interventions and would enhance evidence-based IO practice.

## Introduction

In recent decades, notable progress in immunotherapy has revolutionized cancer therapy. Immunotherapy has been approved for treating various cancers, especially but not only for patients with advanced, recurrent, and metastatic malignancy (1, 2). The immune checkpoint inhibitors (ICI) for immunotherapies include those against programmed cell death protein 1 (PD-1: nivolumab, pembrolizumab, camrelizumab, sintilimab, tislelizumab, cemiplimab, spartalizumab), programmed death-ligand 1 (PD-L1: atezolizumab, avelumab, durvalumab), and cytotoxic T lymphocyte antigen 4 (CTLA-4: ipilimumab, tremelimumab) (3, 4).

Randomized clinical trials (RCTs) are considered the gold standard for assessing medical interventions, especially in antitumor drugs. Publications in peer-reviewed journals are major sources from which clinicians seek to understand clinical trial designs and results. Oncologists use these findings to formulate antitumor treatment regimens, predict the risks and benefits of various treatment options, and improve efficacy. The primary results of RCTs are typically response to the interventions or survival, and harms to the participants. In publications of clinical trials, harm reporting is as important as efficacy reporting. Both are essential for estimating the balance of the benefits and harms of medical measures. Scrutiny of the data shows that the effectiveness is often well explored and documented, whereas the harm is less so (5). Clinicians mainly pay close attention to the benefits rather than adverse outcomes. This is unfortunate, as the safety and harm of a medication are almost as important as its efficacy, especially when a therapeutic decision has to be made for patients with malignant tumors. Moreover, the accuracy and comprehensiveness of immunotherapy-specific immune-related adverse events (irAE) reports are more crucial for determining immunotherapy options in patients. Therefore, a unified standard is needed to ensure the quality of the harm reports in RCTs.

The Consolidated Standards of Reporting Trials (CONSORT) statement is a widely endorsed document that provides a checklist of items that should be included in RCT reports (6). In 2004, the CONSORT group extended this guidance, providing a panel of 10 specific recommendations on harm reporting, with accompanying explanations and examples of appropriate reporting (7). We performed the present study to evaluate the quality of adverse events (AE) reporting in immuno-oncology (IO) clinical trial publications adhering to the CONSORT extension. We also investigated the article characteristics associated with higher quality in IO trial reporting.

## Materials and Methods

### Trial Selection

Citations from the PubMed, Embase, Cochrane Library, and Web of Science databases between January 1, 2010, and December 31, 2021, were reviewed to identify eligible publications for the analysis. The keywords used were: cancer (cancers, carcinoma, neoplasia, neoplasias, neoplasm, tumor, tumors, malignancy, malignancies, leukemia, lymphoma, melanoma, glioma); immune checkpoint inhibitor (immune checkpoint blocking agent, immune therapy, immunotherapy, immunotherapies, immuno-oncology treatment, anti-programmed cell death protein, anti-programmed cell death protein 1, PD-1, PD1, anti-programmed death-ligand 1, PD-L1, PDL1, anti-cytotoxic T-lymphocyte antigen 4, CTLA-4, CTLA4, ipilimumab, tremelimumab, nivolumab, pembrolizumab, camrelizumab, sintilimab, tislelizumab, cemiplimab, spartalizumab, atezolizumab, avelumab, durvalumab); compare (comparison, comparative, comparing). The filters were: article type = clinical trial; language = English; and species = humans.

Only head-to-head phase II and III RCTs involving patients with cancer and comparing ≥2 treatments that included ICI drugs or comparing an ICI drug with conventional therapy were eligible. Phase I studies, observational studies, case reports, review, editorials, letters, registration information, clinical trial protocols, conference abstracts, posters, cost-effectiveness studies, and trials that compared different doses or usages of one ICI drug were excluded, as were secondary analyses or subset analyses of previously published trials. Where multiple publications were identified from the same trial, the initial publication was used for the analysis.

### Data Extraction

Two of the authors (Yuhong Wang and Shaodong Hong) defined a harm reporting quality score (HRQS) based on the 2004 CONSORT extension. The authors reviewed the CONSORT extension guidelines on harms and extracted the specific reporting elements. Sixteen key reporting elements derived from the 10 recommendations were identified (**Table 1**). Each element enrolled in the HRQS was scored 1 if it was adequately reported in the main text or appendix, or 0 if it was not clearly reported or not reported at all. Each element was weighted with equal importance. For recommendations with several subcomponents, a score of 1 was awarded if any one of them was reported. The ninth recommendation, i.e., “describe any subgroup analyses and exploratory analyses for harms,” was excluded, as this element would only apply to the subset of trials that included such subgroups.

**Table 1:** Elements of Harm Reporting.

Article section	2004 CONSORT Recommendation	Elements included in current study	
Title/Abstract	1. Title or abstract states whether harms are addressed in study.	Harms addressed in the title.	1
		Harms addressed in the abstract.	2
Introduction	2. If the trial addresses both harms and benefits, the introduction should so state.	Information on harms addressed in introduction.	3
Methods	3. List addressed adverse events with definitions for each.	Article reported use of a validated instrument/scale for harms.	4
		Article reported the definition of harms.	5
	4. Clarify how harms-related information was collected.	Description of how to collect information on harms	6
		Description of when harms information was collected	7
		Description of stopping rules because of harms	8
	5. Describe plans for presenting and analyzing information on harms.	Article reported the methods for analyzing harms.	9
Results	6. Describe for each arm the participant withdrawals that are a result of harms and the experience with the allocated treatment.	Article reported reasons and number for discontinuation caused by harms.	10
		Article reported reasons and number for death caused by harms.	11
	7. Provide the denominators for analyses on harms.	Article reported absolute numbers of harms.	12
		Article reported which patients were evaluable for toxicity.	13
	8. Present the absolute risk of each adverse event.	Harm results presented separately for each study group.	14
		Severe events presented separately for each type of event.	15
	9. Describe any subgroup analyses and exploratory analyses for harms.	Not included in current analysis^#^	
Discussion	10. Provide a balanced discussion of benefits and harms.	Article provided a balanced view of benefits and harms.	16

CONSORT, Consolidated Standards of Reporting Trials.

^#^Not included in the current analysis as the element would only apply to the subset of trials that included such subgroups.

Trial characteristics that could affect the quality score were extracted from each manuscript, and included publication year, journal type and impact factor, trial phase, funding source, region in which the trial was conducted, the principal investigator’s continent of origin, number of participating centers, sample size, tumor type, mechanism of ICI, treatment strategy, and whether the primary study end point was met. The publication year was directly extracted as a continuous variable. Journal types were classified as oncologic and comprehensive journals according to the types of published articles. Trials were considered industry-funded if they received any form of industry funding. Trials were considered intercontinental when patients from >1 continent were recruited. The principal investigators were from North America, Europe, Asia, or Oceania. Regarding the mechanism, ICI agent in immunotherapy was anti-PD-1, anti-PD-L1, anti-CTLA-4, or any mix thereof (multi-agent). Regarding the treatment strategy, immunotherapy could be used alone (monotherapy or combination of two types of ICI) or combined with other intervention types. The Results section of each publication was examined to determine whether the primary end point was met.

### Statistical Analysis

The HRQS was calculated as the sum of the number of reporting elements (0-16) that were identified from each publication. The HRQS was described using the mean and range. The following variables were considered candidate factors affecting the HRQ in univariate and multivariate linear regression analyses: publication year, journal type, journal impact factor, phase of the trial, trial funding sources, region in which the trial was performed, the principal investigator’s continent of origin, participating centers, sample size, tumor type, ICI agent, immunotherapy strategy, and results of the primary outcome. Considering that potential confounding factors might conceal the implications between trial characteristics and some HRQS-associated variables in univariate analyses, we applied a higher threshold of statistical significance (*P* = 0.2) for entering variables in the multivariable model. As a result, variables with a *P*-value ≤ 0.2 in univariate analyses were selected to enter simultaneously into the forced entry multivariable linear regression model. A multivariable regression model was constructed with the outcome in question as the dependent variable and with potential predictors as the independent variables. All analyses were performed using R software (http://www.R-project.org/). All tests were 2-sided, with *P* < 0.05 considered statistically significant.

## Results

### Characteristics of the Trials

**Figure 1:**
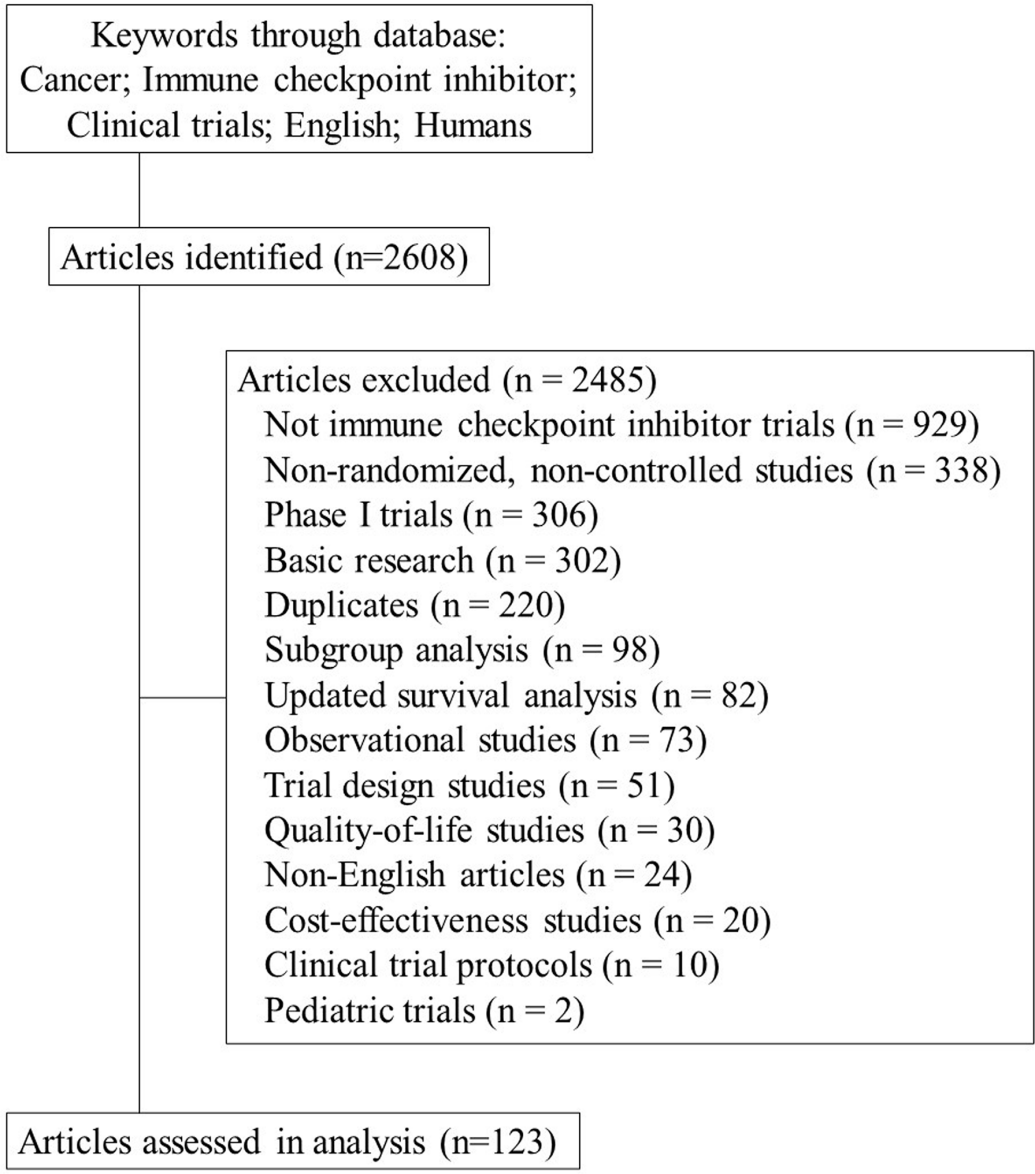
Flowchart of screening of publications on immuno-oncology clinical trial included in the systematic review.

From the 2608 trials initially screened, a total of 123 publications were included in this analysis. The selection process and reasons for exclusion are shown in **Figure 1**. **Table 2** lists the characteristics of the publications included in the analysis.

**Table 2:** Characteristics of trials included in analysis.

Characteristic	Trials (N = 123)	
	No.	%
**Year of publication**		
2010	1	1
2011	2	2
2012	1	1
2013	2	2
2014	2	2
2015	9	7
2016	8	7
2017	9	7
2018	18	15
2019	17	14
2020	11	9
2021	43	35
**Journal**		
The New England Journal of Medicine	36	29
Lancet Oncology	25	20
The Lancet	20	16
Journal of Clinical Oncology	14	11
Annals of Oncology	8	7
Journal of Thoracic Oncology	6	5
Jama Oncology	4	3
Clinical Cancer Research	3	2
Jama	2	2
Nature Medicine	1	1
The Lancet Respiratory Medicine	1	1
Journal for ImmunoTherapy of Cancer	1	1
European Journal of Cancer	1	1
Investigational New Drugs	1	1
**Journal impact factor**		
<30	12	10
30~60	55	45
>60	56	46
**Phase of trial**		
Phase II	24	20
Phase III	99	80
**Sources of trial funding**		
Funded by industry	121	98
No industry funding	2	2
**Region in which trial was led**		
International	104	85
Asia	11	9
North America	4	3
Europe	3	2
Oceania	1	1
**Continent where is the principal investigator from**		
North America	64	52
Europe	33	27
Asia	23	19
Oceania	3	2
**Participating centers, No. of centers**		
Median	127	
Interquartile range	3-246	
Unknow	20	
**Sample size, No. of patients**		
Median	566	
Interquartile range	72-1739	
**Tumor type**		
Lung cancer	43	35
Melanoma	22	18
Esophageal or gastric or gastro-oesophageal junction cancer	13	11
Renal cell carcinoma	9	7
Urothelial Carcinoma	7	6
Breast Cancer	6	5
Head and neck squamous cell carcinoma	5	4
Hepatocellular carcinoma	4	3
Ovarian cancer	4	3
Malignant mesothelioma	3	2
Colorectal cancer	2	2
Prostate cancer	2	2
Nasopharyngeal carcinoma	1	1
Cervical cancer	1	1
Hodgkin lymphoma	1	1
**Immune checkpoint blocking agent**		
Anti-PD-1	60	49
Anti-PD-L1	31	25
Anti-CTLA-4	16	13
Multi-agents	16	13
Anti-PD-1+ Anti-CTLA-4	14	11
Anti-PD-L1+ Anti-CTLA-4	2	2
**Immunotherapy strategy**		
Immunotherapy (monotherapy or combination of two types of ICI)	77	63
Combined with other intervention type	46	37
Chemotherapy	30	24
Target therapy	10	8
Others	6	5
**Trial met primary end point**		
Yes	80	65
No	43	35

PD-1, programmed cell death protein 1; PD-L1, programmed death-ligand 1; CTLA-4, cytotoxic T-lymphocyte antigen 4; ICI, immune checkpoint inhibitors.

The number of published RCTs increased slowly from 2010 to 2017 per year, then increased dramatically after 2018, especially in 2021. Nearly half of the trials (56/123, 46%) were published in journals with impact factor > 60, including *The New England Journal of Medicine* (36/123, 29%), *The Lancet* (20/123, 16%), and *JAMA* (2/123, 2%). Most of the RCTs were intercontinental (104/123, 85%) and 52% of the principal investigators were from North America (64/123). The majority of the trials (121/123, 98%) were industry-funded. The most common tumor type examined was lung cancer (43/123, 35%), followed by melanoma (22/123, 18%) and renal cell carcinoma (9/123, 7%). The main treatment strategy was immunotherapy alone (monotherapy or combination of two types of ICI; 77/123, 63%). Anti-PD-1 was the most frequently used ICI agent (60/123, 49%).

### Quality Score According to CONSORT Statement

**Figure 2:**
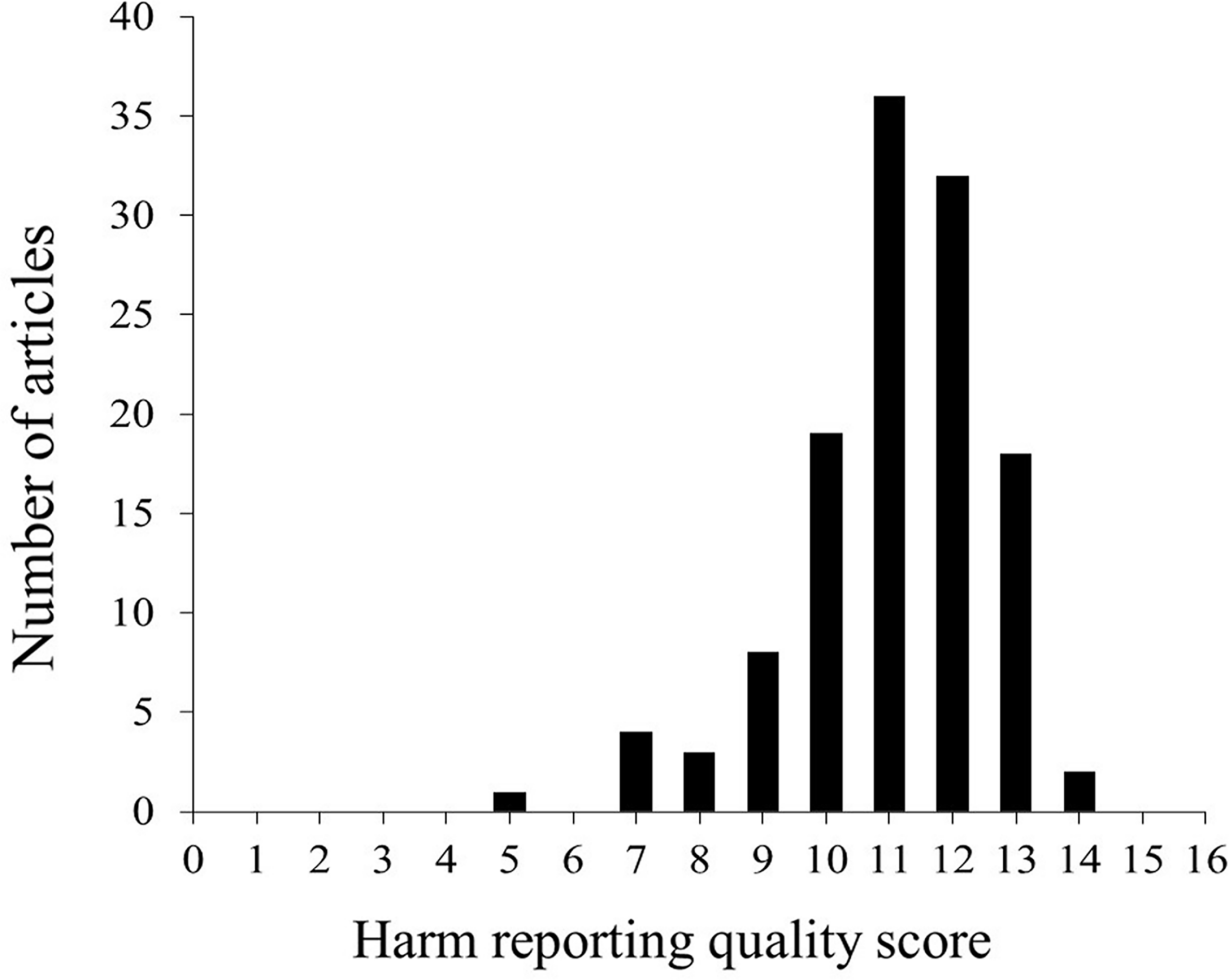
Distribution of harm reporting quality scores.

On a 16-point scale, the mean HRQS for all elements was 11.1 (range, 5-14; **Figure 2**). Most of the publications were scored 10-13 (105/123, 85%), while five publications (7%) had scores ≤ 8. No publication had a score of 16.

Certain reporting elements were more consistently addressed in the publications. The majority of publications stated the harms in the abstract (item 2, 99%), specified the instrument/scale used for classifying AE (item 4, 98%), reported absolute numbers of AE (item 12, 99%), presented the harm results separately for each study group (item 14, 99%), and presented severe events separately for each type of event (item 15, 98%). However, only two publications addressed harms in the title (item 1, 2%). Nearly half of the publications stated harms information in the introduction section (item 3, 46%). Items pertaining to the methods of AE information collection and analysis were poorly reported. Specifically, only 3% of publications reported adequately on how information on harms was collected (item 6), while only 11% adequately described the methods for analyzing harms (item 9). Up to 47% of the publications stated when harms information was collected (item 7) and 60% of publications clearly defined the AE (item 5). **Table 3** details these findings.

**Table 3:** Quality of harms reporting using each 16 adverse event reporting elements.

Harms reporting elements	Trials in which item was adequately reported	
	No.	%
1. Harms addressed in the title.	2	2
2. Harms addressed in the abstract.	122	99
3. Information on harms addressed in introduction.	56	46
4. Article reported use of a validated instrument/scale for harms.	120	98
5. Article reported the definition of harms.	74	60
6. Description of how to collect information on harms.	4	3
7. Description of when harms information was collected.	58	47
8. Description of stopping rules because of harms.	104	85
9. Article reported the methods for analyzing harms.	13	11
10.Article reported reasons and number for discontinuation caused by harms.	111	90
11.Article reported reasons and number for death caused by harms.	111	90
12.Article reported absolute numbers of harms.	122	99
13.Article reported which patients were evaluable for toxicity.	109	89
14.Harm results presented separately for each study group.	122	99
15.Severe events presented separately for each type of event.	120	98
16.Article provided a balanced view of benefits and harms.	133	92

### Presentation of Immune-Related Adverse Events

The definition of irAE, which were unique to IO therapy, was clearly described in 60% of the publications either in the main text or appendix. The irAE results were reported separately in 89% of the publications, and 78% of the articles reported them in the main text. However, irAE time of onset and duration, and the clinical interventions used for managing irAE were poorly reported, being mentioned only in 10% and 24% of the main texts, respectively. The two elements were reported in 14% and 33% of the main text or appendices, respectively. **Table 4** details these findings.

**Table 4:** Presentation of immune-related adverse events.

Immune-related adverse events reporting elements	No. of Trials (%)		
	In the main text	In the appendix	In the main text or appendix
Article specifies definition of irAE.	49 (40)	70 (57)	74 (60)
Article reports irAE separately	96 (78)	100 (81)	109 (89)
Article reports time of onset and duration of irAE	12 (10)	13 (11)	17 (14)
Article reports clinical interventions used to manage irAE	30 (24)	30 (24)	40 (33)

irAE, immune-related adverse event.

### Characteristics Associated With Reporting Quality

**Table 5** lists the univariable and multivariable linear regression results. In univariate analyses, the following trial characteristics were associated with higher HRQS: journal impact factor (P=0.003), phase of trial (P<0.001), principal investigator’s continent of origin (P=0.032), number of participating centers (P=0.003), and trial met primary outcome (P=0.011).

**Table 5:** Trial Characteristics associated with harm reporting quality score (0-16 scale).

Trial Characteristics	HRQS		Linear Regression					
			Univariate Analysis			Multivariate Analysis		
	Mean	SE	Estimate	SE	P	Estimate	SE	P
**Year of publication, continuous**	—	—	-0.004	0.055	0.937			
**Journal type**								
Oncologic journal	11.1	1.826	Reference		0.822			
Comprehensive journal	11.03	1.264	-0.064	0.284				
**Journal impact factor**								
<30	9.67	2.06	Reference		0.003	Reference		
30~60	11.35	1.647	1.679	0.479		1.192	0.508	0.021
>60	11.09	1.195	1.423	0.479		0.645	0.626	0.305
**Phase of trial**								
Phase II	10	2.303	Reference		<0.001	Reference		
Phase III	11.32	1.211	1.323	0.337		1.030	0.445	0.023
**Sources of trial funding**								
Funded by industry	11.09	1.555	Reference		0.155	Reference		
No industry funding	9.5	2.121	-1.591	1.112		-0.298	1.105	0.788
**Region in which trial was led**								
International	11.09	1.442	Reference		0.723			
Others	10.95	2.172	-0.139	0.392				
**Continent where is the principal investigator from**								
North America	10.81	1.562	Reference		0.032	Reference		
Europe	10.94	1.713	0.127	0.328		0.077	0.309	0.805
Asia	11.78	1.166	0.970	0.372		0.621	0.400	0.123
Oceania	12.33	0.577	1.521	0.903		1.170	0.866	0.180
**Participating centers, No. of centers**								
≤120	11.35	1.591	Reference		0.003	Reference		
>120	11.2	1.325	-0.154	0.297		-0.146	0.347	0.675
Unknown	10	1.747	-1.354	0.401		-0.628	0.416	0.134
**Sample size, continuous**	—	—	0.000	0.000	0.067	0.000	0.000	0.891
**Tumor type**								
Lung cancer	10.77	1.716	Reference		0.402			
Melanoma	11.14	1.356	0.369	0.411				
Urinary system	10.94	1.305	0.177	0.440				
Digestive system	11.21	1.398	0.443	0.432				
Others	11.57	1.777	0.804	0.417				
**Immune checkpoint blocking agent**								
Anti-PD-1	11.33	1.654	Reference		0.183	Reference		
Anti-PD-L1	10.58	1.455	-0.753	0.344		-0.580	0.340	0.091
Anti-CTLA-4	10.94	1.611	-0.396	0.437		-0.456	0.460	0.324
Multi-agents	11.13	1.258	-0.208	0.437		-0.057	0.431	0.895
**Immunotherapy strategy**								
Immunotherapy (monotherapy or combination of two types of ICI)	11.03	1.739	Reference		0.590			
Combined with chemotherapy	11.23	1.104	0.207	0.340				
Combined with target therapy	10.8	1.398	-0.226	0.532				
Combined with others	11.17	1.722	0.141	0.671				
**Results of the primary outcome**								
Positive	11.32	1.145	Reference		0.011	Reference		
Negative	10.58	2.074	-0.744	0.290		-0.602	0.367	0.104

HRQS, harm reporting quality score; SE, standard error; PD-1, programmed cell death protein 1; PD-L1, programmed death-ligand 1; CTLA-4, cytotoxic T-lymphocyte antigen 4; ICI, immune checkpoint inhibitors.

The multivariable regression model subsequently revealed that higher IF (30<IF<60 vs. IF<30, P=0.021), phase III clinical trial (phase III vs. phase II, P=0.023) were independent predictors of higher HRQS. Specifically, publications with 30<IF<60 had a HRQS on average 1.192 points higher than those with IF<30. The HRQS of phase III clinical trials was higher than those phase II by a mean of 1.030 points.

## Discussion

Transparent and comprehensive reporting of harm data in oncology RCT publications is of critical importance, particularly in IO clinical trials as a new therapy intervention. Therefore, standardized AE reporting is essential and important. In the present study, we systematically evaluated the HRQS in IO clinical trials according to the 2004 CONSORT extension statement on AE reporting.

There have been similar evaluations of HRQS in other medical specialties (8–14). Most of these studies did not propose any quality score (12–14). In oncology publications, Sivendran et al. defined a 14-point score based on the CONSORT statement. They evaluated 175 phase III RCTs on metastatic solid malignancies and reported that the median completeness score was 8 (15). Another study reviewed 325 oncology trials and obtained a mean score of 10.1 using a 16-item quality score based on the CONSORT statement (16). In the present study, we obtained a similar mean HRQS of 11.1 based on the specific 16-element scoring system derived from the CONSORT guideline. Most of the publications had scores of 10-13 (85%). Although each quality score item was not identical between studies, the harm reporting in IO trials was generally unsatisfactory.

In the present study, we found that in most publications, harm information was poorly reported in the Methods section (items 4-9), especially AE collection and analysis, which were mentioned in only 3% and 11% of articles, respectively. The definition and collection time of AE were insufficiently detailed in 40% and 53% of articles, respectively. Prior analysis has also shown similar deficiencies in reporting items pertaining to the methods of AE collection and analysis (16). These findings may result from the perception that AE collection and analysis methodology is conventional and homogeneous. In addition, the titles seldom contained the terms “harm” or “adverse event”. Only two reports contained the analogous word “safety” in their titles. This may be due to the word limit for titles in journals, and the primary end points of the assessing publications were mainly efficacy rather than safety. However, “safety” is a reassuring term that may obscure the real and potentially major harms that any interventions may cause. The members of the CONSORT Group encourage authors to use the term “harms” instead of “safety” (7).

Although the AE reporting in the Methods section of the publications was insufficient, we found that harm information was adequately described in the Results and Discussion sections. Here, 89-99% of the publications provided the absolute numbers of harms, presented harms separately for each study group and each type of event, and stated the reasons for discontinuations and death caused by harms. In the Discussion section, 92% of the articles appraised data on the benefits and harms, and contrasted the trial results on harms with other sources of information on harms.

Compared with the toxicities caused by conventional therapy (chemotherapy, targeted therapy, and their combinations), the irAE from ICI drugs are unique in terms of the organs involved, onset patterns, severity, and management (17). Therefore, detailed reports of irAE are very important for clinicians to understand the manifestations and management of harms in IO clinical trials. In the present study, 89% of the publications presented the irAE outcomes separately for each study group, with separate information on the severity grade of the event, if relevant. Seventy-four publications (60%) defined irAE relatively well; the irAE were described in the main text of 49 articles. The remaining publications mostly used categories such as “immune-related adverse events,” “immune-mediated adverse events,” “treatment-related select adverse events,” or “adverse events of special interest” without defining them. Moreover, we noted that the irAE time of onset and duration, and management were poorly reported (reported in 10% and 24% of articles, respectively) in the main text. Even including the appendix, the reporting adequacy was raised to only 14% and 33%, respectively. These findings indicate that researchers usually focus on the manifestations of irAE rather than the onset patterns and management. Given that the toxicity of immunotherapy can be of latent occurrence and long-lasting (18, 19), reporting irAE onset patterns and management is arguably as clinically important for evaluating the risk–benefit and helpful for optimizing the design of future trials.

In the present study, we investigated the factors associated with higher HRQS. The publications in journals of 30<IF<60 had a higher quality score than journals of IF<30. This finding is similar to the results of previous study (20) and may be explained by stricter peer review or higher scrutiny before submission to higher IF journals. We also found that the HRQS was higher for phase III clinical trials compared with phase II trials. The possible reason is that considering more participants in phase III clinical trials and the higher requirements concerning the monitoring of participants, the researchers reported AE in more detail.

The present study has several limitations. First, the assessment was restricted to randomized phase II and III IO clinical trials and did not take into account the harm reporting in cohort or observational studies, in which it is more appropriate to report mid-term and long-term safety. Second, as we restricted our analysis to phase II and III trials involving solid and hematologic malignancy, it resulted in a small number of publications being enrolled and impacted the credibility of the statistical results to a certain extent. Further, whether each of the recommendations outlined in the CONSORT extension is of equal importance, or even practical, may be controversial. Here, we gave equal weight to each element and subcomponent, which may have weakened some important elements or overemphasized some less important elements.

In conclusion, our findings suggest that, according to the 2004 CONSORT extension statement, the AE reporting quality in IO trials is suboptimal. The methodological aspects of AE collection and analysis, and the irAE onset patterns and management, are often poorly reported. Efforts should be made to better describe AE and to standardize reporting practices. High-quality trials focusing on AE are required to aid clinicians in improving early management and recognition of irAE.

## Data Availability Statement

The original contributions presented in the study are included in the article/**Supplementary Material**. Further inquiries can be directed to the corresponding authors.

## Author Contributions

All authors contributed to the study conception and design. Data acquisition, data interpretation and statistical analysis were performed by YW, CC, and WD. The first draft of the manuscript was written by YW, and all authors commented on previous versions of the manuscript. SH and LZ contributed to the study design and statistical analysis. All authors read and approved the final manuscript.

## Conflict of Interest

The authors declare that the research was conducted in the absence of any commercial or financial relationships that could be construed as a potential conflict of interest.

## Publisher’s Note

All claims expressed in this article are solely those of the authors and do not necessarily represent those of their affiliated organizations, or those of the publisher, the editors and the reviewers. Any product that may be evaluated in this article, or claim that may be made by its manufacturer, is not guaranteed or endorsed by the publisher.
