# Supplementary material for: Adverse Event Reporting Quality in Cancer Clinical Trials Evaluating Immune Checkpoint Inhibitor Therapy: A Systematic Review
Source: Front Immunol. 2022 Jul 7;13:874829. doi: 10.3389/fimmu.2022.874829 (PMC9301013; doi:10.3389/fimmu.2022.874829)
Supplement: Supplementary file 1 [file DataSheet_1.docx]

***Supplementary Material***

**The List of Immuno-oncology clinical trial reports included in this study：**

[1] Hodi F S, O'Day S J, McDermott D F, et al. Improved survival with ipilimumab in patients with metastatic melanoma[J]. N Engl J Med, 2010,363(8):711-723.

[2] Hersh E M, O'Day S J, Powderly J, et al. A phase II multicenter study of ipilimumab with or without dacarbazine in chemotherapy-naive patients with advanced melanoma[J]. Invest New Drugs, 2011,29(3):489-498.

[3] Robert C, Thomas L, Bondarenko I, et al. Ipilimumab plus dacarbazine for previously untreated metastatic melanoma[J]. N Engl J Med, 2011,364(26):2517-2526.

[4] Lynch T J, Bondarenko I, Luft A, et al. Ipilimumab in combination with paclitaxel and carboplatin as first-line treatment in stage IIIB/IV non-small-cell lung cancer: results from a randomized, double-blind, multicenter phase II study[J]. J Clin Oncol, 2012,30(17):2046-2054.

[5] Reck M, Bondarenko I, Luft A, et al. Ipilimumab in combination with paclitaxel and carboplatin as first-line therapy in extensive-disease-small-cell lung cancer: results from a randomized, double-blind, multicenter phase 2 trial[J]. Ann Oncol, 2013,24(1):75-83.

[6] Ribas A, Kefford R, Marshall M A, et al. Phase III randomized clinical trial comparing tremelimumab with standard-of-care chemotherapy in patients with advanced melanoma[J]. J Clin Oncol, 2013,31(5):616-622.

[7] Hodi F S, Lee S, McDermott D F, et al. Ipilimumab plus sargramostim vs ipilimumab alone for treatment of metastatic melanoma: a randomized clinical trial[J]. JAMA, 2014,312(17):1744-1753.

[8] Kwon E D, Drake C G, Scher H I, et al. Ipilimumab versus placebo after radiotherapy in patients with metastatic castration-resistant prostate cancer that had progressed after docetaxel chemotherapy (CA184-043): a multicentre, randomised, double-blind, phase 3 trial[J]. Lancet Oncol, 2014,15(7):700-712.

[9] Brahmer J, Reckamp K L, Baas P, et al. Nivolumab versus Docetaxel in Advanced Squamous-Cell Non-Small-Cell Lung Cancer[J]. N Engl J Med, 2015,373(2):123-135.

[10] Borghaei H, Paz-Ares L, Horn L, et al. Nivolumab versus Docetaxel in Advanced Nonsquamous Non-Small-Cell Lung Cancer[J]. N Engl J Med, 2015,373(17):1627-1639.

[11] Weber J S, D'Angelo S P, Minor D, et al. Nivolumab versus chemotherapy in patients with advanced melanoma who progressed after anti-CTLA-4 treatment (CheckMate 037): a randomised, controlled, open-label, phase 3 trial[J]. Lancet Oncol, 2015,16(4):375-384.

[12] Robert C, Long G V, Brady B, et al. Nivolumab in previously untreated melanoma without BRAF mutation[J]. N Engl J Med, 2015,372(4):320-330.

[13] Larkin J, Chiarion-Sileni V, Gonzalez R, et al. Combined Nivolumab and Ipilimumab or Monotherapy in Untreated Melanoma[J]. N Engl J Med, 2015,373(1):23-34.

[14] Eggermont A M, Chiarion-Sileni V, Grob J J, et al. Adjuvant ipilimumab versus placebo after complete resection of high-risk stage III melanoma (EORTC 18071): a randomised, double-blind, phase 3 trial[J]. Lancet Oncol, 2015,16(5):522-530.

[15] Ribas A, Puzanov I, Dummer R, et al. Pembrolizumab versus investigator-choice chemotherapy for ipilimumab-refractory melanoma (KEYNOTE-002): a randomised, controlled, phase 2 trial[J]. Lancet Oncol, 2015,16(8):908-918.

[16] Robert C, Schachter J, Long G V, et al. Pembrolizumab versus Ipilimumab in Advanced Melanoma[J]. N Engl J Med, 2015,372(26):2521-2532.

[17] Motzer R J, Escudier B, McDermott D F, et al. Nivolumab versus Everolimus in Advanced Renal-Cell Carcinoma[J]. N Engl J Med, 2015,373(19):1803-1813.

[18] Postow M A, Chesney J, Pavlick A C, et al. Nivolumab and ipilimumab versus ipilimumab in untreated melanoma[J]. N Engl J Med, 2015,372(21):2006-2017.

[19] Langer C J, Gadgeel S M, Borghaei H, et al. Carboplatin and pemetrexed with or without pembrolizumab for advanced, non-squamous non-small-cell lung cancer: a randomised, phase 2 cohort of the open-label KEYNOTE-021 study[J]. Lancet Oncol, 2016,17(11):1497-1508.

[20] Reck M, Rodriguez-Abreu D, Robinson A G, et al. Pembrolizumab versus Chemotherapy for PD-L1-Positive Non-Small-Cell Lung Cancer[J]. N Engl J Med, 2016,375(19):1823-1833.

[21] Fehrenbacher L, Spira A, Ballinger M, et al. Atezolizumab versus docetaxel for patients with previously treated non-small-cell lung cancer (POPLAR): a multicentre, open-label, phase 2 randomised controlled trial[J]. Lancet, 2016,387(10030):1837-1846.

[22] Ferris R L, Blumenschein G J, Fayette J, et al. Nivolumab for Recurrent Squamous-Cell Carcinoma of the Head and Neck[J]. N Engl J Med, 2016,375(19):1856-1867.

[23] Reck M, Luft A, Szczesna A, et al. Phase III Randomized Trial of Ipilimumab Plus Etoposide and Platinum Versus Placebo Plus Etoposide and Platinum in Extensive-Stage Small-Cell Lung Cancer[J]. J Clin Oncol, 2016,34(31):3740-3748.

[24] Herbst R S, Baas P, Kim D W, et al. Pembrolizumab versus docetaxel for previously treated, PD-L1-positive, advanced non-small-cell lung cancer (KEYNOTE-010): a randomised controlled trial[J]. Lancet, 2016,387(10027):1540-1550.

[25] Govindan R, Szczesna A, Ahn M J, et al. Phase III Trial of Ipilimumab Combined With Paclitaxel and Carboplatin in Advanced Squamous Non-Small-Cell Lung Cancer[J]. J Clin Oncol, 2017,35(30):3449-3457.

[26] Beer T M, Kwon E D, Drake C G, et al. Randomized, Double-Blind, Phase III Trial of Ipilimumab Versus Placebo in Asymptomatic or Minimally Symptomatic Patients With Metastatic Chemotherapy-Naive Castration-Resistant Prostate Cancer[J]. J Clin Oncol, 2017,35(1):40-47.

[27] Maio M, Scherpereel A, Calabro L, et al. Tremelimumab as second-line or third-line treatment in relapsed malignant mesothelioma (DETERMINE): a multicentre, international, randomised, double-blind, placebo-controlled phase 2b trial[J]. Lancet Oncol, 2017,18(9):1261-1273.

[28] Carbone D P, Reck M, Paz-Ares L, et al. First-Line Nivolumab in Stage IV or Recurrent Non-Small-Cell Lung Cancer[J]. N Engl J Med, 2017,376(25):2415-2426.

[29] Rittmeyer A, Barlesi F, Waterkamp D, et al. Atezolizumab versus docetaxel in patients with previously treated non-small-cell lung cancer (OAK): a phase 3, open-label, multicentre randomised controlled trial[J]. Lancet, 2017,389(10066):255-265.

[30] Antonia S J, Villegas A, Daniel D, et al. Durvalumab after Chemoradiotherapy in Stage III Non-Small-Cell Lung Cancer[J]. N Engl J Med, 2017,377(20):1919-1929.

[31] Bellmunt J, de Wit R, Vaughn D J, et al. Pembrolizumab as Second-Line Therapy for Advanced Urothelial Carcinoma[J]. N Engl J Med, 2017,376(11):1015-1026.

[32] Kang Y K, Boku N, Satoh T, et al. Nivolumab in patients with advanced gastric or gastro-oesophageal junction cancer refractory to, or intolerant of, at least two previous chemotherapy regimens (ONO-4538-12, ATTRACTION-2): a randomised, double-blind, placebo-controlled, phase 3 trial[J]. Lancet, 2017,390(10111):2461-2471.

[33] Bang Y J, Cho J Y, Kim Y H, et al. Efficacy of Sequential Ipilimumab Monotherapy versus Best Supportive Care for Unresectable Locally Advanced/Metastatic Gastric or Gastroesophageal Junction Cancer[J]. Clin Cancer Res, 2017,23(19):5671-5678.

[34] Weber J, Mandala M, Del V M, et al. Adjuvant Nivolumab versus Ipilimumab in Resected Stage III or IV Melanoma[J]. N Engl J Med, 2017,377(19):1824-1835.

[35] Hellmann M D, Ciuleanu T E, Pluzanski A, et al. Nivolumab plus Ipilimumab in Lung Cancer with a High Tumor Mutational Burden[J]. N Engl J Med, 2018,378(22):2093-2104.

[36] Horn L, Mansfield A S, Szczesna A, et al. First-Line Atezolizumab plus Chemotherapy in Extensive-Stage Small-Cell Lung Cancer[J]. N Engl J Med, 2018,379(23):2220-2229.

[37] Socinski M A, Jotte R M, Cappuzzo F, et al. Atezolizumab for First-Line Treatment of Metastatic Nonsquamous NSCLC[J]. N Engl J Med, 2018,378(24):2288-2301.

[38] Barlesi F, Vansteenkiste J, Spigel D, et al. Avelumab versus docetaxel in patients with platinum-treated advanced non-small-cell lung cancer (JAVELIN Lung 200): an open-label, randomised, phase 3 study[J]. Lancet Oncol, 2018,19(11):1468-1479.

[39] Paz-Ares L, Luft A, Vicente D, et al. Pembrolizumab plus Chemotherapy for Squamous Non-Small-Cell Lung Cancer[J]. N Engl J Med, 2018,379(21):2040-2051.

[40] Gandhi L, Rodriguez-Abreu D, Gadgeel S, et al. Pembrolizumab plus Chemotherapy in Metastatic Non-Small-Cell Lung Cancer[J]. N Engl J Med, 2018,378(22):2078-2092.

[41] Eggermont A, Blank C U, Mandala M, et al. Adjuvant Pembrolizumab versus Placebo in Resected Stage III Melanoma[J]. N Engl J Med, 2018,378(19):1789-1801.

[42] Chesney J, Puzanov I, Collichio F, et al. Randomized, Open-Label Phase II Study Evaluating the Efficacy and Safety of Talimogene Laherparepvec in Combination With Ipilimumab Versus Ipilimumab Alone in Patients With Advanced, Unresectable Melanoma[J]. J Clin Oncol, 2018,36(17):1658-1667.

[43] Long G V, Atkinson V, Lo S, et al. Combination nivolumab and ipilimumab or nivolumab alone in melanoma brain metastases: a multicentre randomised phase 2 study[J]. Lancet Oncol, 2018,19(5):672-681.

[44] Powles T, Duran I, van der Heijden M S, et al. Atezolizumab versus chemotherapy in patients with platinum-treated locally advanced or metastatic urothelial carcinoma (IMvigor211): a multicentre, open-label, phase 3 randomised controlled trial[J]. Lancet, 2018,391(10122):748-757.

[45] Schmid P, Adams S, Rugo H S, et al. Atezolizumab and Nab-Paclitaxel in Advanced Triple-Negative Breast Cancer[J]. N Engl J Med, 2018,379(22):2108-2121.

[46] Motzer R J, Tannir N M, McDermott D F, et al. Nivolumab plus Ipilimumab versus Sunitinib in Advanced Renal-Cell Carcinoma[J]. N Engl J Med, 2018,378(14):1277-1290.

[47] McDermott D F, Huseni M A, Atkins M B, et al. Clinical activity and molecular correlates of response to atezolizumab alone or in combination with bevacizumab versus sunitinib in renal cell carcinoma[J]. Nat Med, 2018,24(6):749-757.

[48] Bang Y J, Ruiz E Y, Van Cutsem E, et al. Phase III, randomised trial of avelumab versus physician's choice of chemotherapy as third-line treatment of patients with advanced gastric or gastro-oesophageal junction cancer: primary analysis of JAVELIN Gastric 300[J]. Ann Oncol, 2018,29(10):2052-2060.

[49] Shitara K, Ozguroglu M, Bang Y J, et al. Pembrolizumab versus paclitaxel for previously treated, advanced gastric or gastro-oesophageal junction cancer (KEYNOTE-061): a randomised, open-label, controlled, phase 3 trial[J]. Lancet, 2018,392(10142):123-133.

[50] Tarhini A A, Lee S J, Li X, et al. E3611-A Randomized Phase II Study of Ipilimumab at 3 or 10 mg/kg Alone or in Combination with High-Dose Interferon-alpha2b in Advanced Melanoma[J]. Clin Cancer Res, 2019,25(2):524-532.

[51] Levy B P, Giaccone G, Besse B, et al. Randomised phase 2 study of pembrolizumab plus CC-486 versus pembrolizumab plus placebo in patients with previously treated advanced non-small cell lung cancer[J]. Eur J Cancer, 2019,108:120-128.

[52] Siu L L, Even C, Mesia R, et al. Safety and Efficacy of Durvalumab With or Without Tremelimumab in Patients With PD-L1-Low/Negative Recurrent or Metastatic HNSCC: The Phase 2 CONDOR Randomized Clinical Trial[J]. JAMA Oncol, 2019,5(2):195-203.

[53] Paz-Ares L, Dvorkin M, Chen Y, et al. Durvalumab plus platinum-etoposide versus platinum-etoposide in first-line treatment of extensive-stage small-cell lung cancer (CASPIAN): a randomised, controlled, open-label, phase 3 trial[J]. Lancet, 2019,394(10212):1929-1939.

[54] Wu Y L, Lu S, Cheng Y, et al. Nivolumab Versus Docetaxel in a Predominantly Chinese Patient Population With Previously Treated Advanced NSCLC: CheckMate 078 Randomized Phase III Clinical Trial[J]. J Thorac Oncol, 2019,14(5):867-875.

[55] Hellmann M D, Paz-Ares L, Bernabe C R, et al. Nivolumab plus Ipilimumab in Advanced Non-Small-Cell Lung Cancer[J]. N Engl J Med, 2019,381(21):2020-2031.

[56] West H, McCleod M, Hussein M, et al. Atezolizumab in combination with carboplatin plus nab-paclitaxel chemotherapy compared with chemotherapy alone as first-line treatment for metastatic non-squamous non-small-cell lung cancer (IMpower130): a multicentre, randomised, open-label, phase 3 trial[J]. Lancet Oncol, 2019,20(7):924-937.

[57] Mok T, Wu Y L, Kudaba I, et al. Pembrolizumab versus chemotherapy for previously untreated, PD-L1-expressing, locally advanced or metastatic non-small-cell lung cancer (KEYNOTE-042): a randomised, open-label, controlled, phase 3 trial[J]. Lancet, 2019,393(10183):1819-1830.

[58] Theelen W, Peulen H, Lalezari F, et al. Effect of Pembrolizumab After Stereotactic Body Radiotherapy vs Pembrolizumab Alone on Tumor Response in Patients With Advanced Non-Small Cell Lung Cancer: Results of the PEMBRO-RT Phase 2 Randomized Clinical Trial[J]. JAMA Oncol, 2019,5(9):1276-1282.

[59] Lebbe C, Meyer N, Mortier L, et al. Evaluation of Two Dosing Regimens for Nivolumab in Combination With Ipilimumab in Patients With Advanced Melanoma: Results From the Phase IIIb/IV CheckMate 511 Trial[J]. J Clin Oncol, 2019,37(11):867-875.

[60] Long G V, Dummer R, Hamid O, et al. Epacadostat plus pembrolizumab versus placebo plus pembrolizumab in patients with unresectable or metastatic melanoma (ECHO-301/KEYNOTE-252): a phase 3, randomised, double-blind study[J]. Lancet Oncol, 2019,20(8):1083-1097.

[61] Eng C, Kim T W, Bendell J, et al. Atezolizumab with or without cobimetinib versus regorafenib in previously treated metastatic colorectal cancer (IMblaze370): a multicentre, open-label, phase 3, randomised, controlled trial[J]. Lancet Oncol, 2019,20(6):849-861.

[62] Loibl S, Untch M, Burchardi N, et al. A randomised phase II study investigating durvalumab in addition to an anthracycline taxane-based neoadjuvant therapy in early triple-negative breast cancer: clinical results and biomarker analysis of GeparNuevo study[J]. Ann Oncol, 2019,30(8):1279-1288.

[63] Rini B I, Powles T, Atkins M B, et al. Atezolizumab plus bevacizumab versus sunitinib in patients with previously untreated metastatic renal cell carcinoma (IMmotion151): a multicentre, open-label, phase 3, randomised controlled trial[J]. Lancet, 2019,393(10189):2404-2415.

[64] Motzer R J, Penkov K, Haanen J, et al. Avelumab plus Axitinib versus Sunitinib for Advanced Renal-Cell Carcinoma[J]. N Engl J Med, 2019,380(12):1103-1115.

[65] Rini B I, Plimack E R, Stus V, et al. Pembrolizumab plus Axitinib versus Sunitinib for Advanced Renal-Cell Carcinoma[J]. N Engl J Med, 2019,380(12):1116-1127.

[66] Kato K, Cho B C, Takahashi M, et al. Nivolumab versus chemotherapy in patients with advanced oesophageal squamous cell carcinoma refractory or intolerant to previous chemotherapy (ATTRACTION-3): a multicentre, randomised, open-label, phase 3 trial[J]. Lancet Oncol, 2019,20(11):1506-1517.

[67] Cohen E, Soulieres D, Le Tourneau C, et al. Pembrolizumab versus methotrexate, docetaxel, or cetuximab for recurrent or metastatic head-and-neck squamous cell carcinoma (KEYNOTE-040): a randomised, open-label, phase 3 study[J]. Lancet, 2019,393(10167):156-167.

[68] Burtness B, Harrington K J, Greil R, et al. Pembrolizumab alone or with chemotherapy versus cetuximab with chemotherapy for recurrent or metastatic squamous cell carcinoma of the head and neck (KEYNOTE-048): a randomised, open-label, phase 3 study[J]. Lancet, 2019,394(10212):1915-1928.

[69] Finn R S, Ryoo B Y, Merle P, et al. Pembrolizumab As Second-Line Therapy in Patients With Advanced Hepatocellular Carcinoma in KEYNOTE-240: A Randomized, Double-Blind, Phase III Trial[J]. J Clin Oncol, 2020,38(3):193-202.

[70] Herbst R S, Giaccone G, de Marinis F, et al. Atezolizumab for First-Line Treatment of PD-L1-Selected Patients with NSCLC[J]. N Engl J Med, 2020,383(14):1328-1339.

[71] Finn R S, Qin S, Ikeda M, et al. Atezolizumab plus Bevacizumab in Unresectable Hepatocellular Carcinoma[J]. N Engl J Med, 2020,382(20):1894-1905.

[72] Zimmer L, Livingstone E, Hassel J C, et al. Adjuvant nivolumab plus ipilimumab or nivolumab monotherapy versus placebo in patients with resected stage IV melanoma with no evidence of disease (IMMUNED): a randomised, double-blind, placebo-controlled, phase 2 trial[J]. Lancet, 2020,395(10236):1558-1568.

[73] Gutzmer R, Stroyakovskiy D, Gogas H, et al. Atezolizumab, vemurafenib, and cobimetinib as first-line treatment for unresectable advanced BRAF(V600) mutation-positive melanoma (IMspire150): primary analysis of the randomised, double-blind, placebo-controlled, phase 3 trial[J]. Lancet, 2020,395(10240):1835-1844.

[74] Andre T, Shiu K K, Kim T W, et al. Pembrolizumab in Microsatellite-Instability-High Advanced Colorectal Cancer[J]. N Engl J Med, 2020,383(23):2207-2218.

[75] Galsky M D, Arija J, Bamias A, et al. Atezolizumab with or without chemotherapy in metastatic urothelial cancer (IMvigor130): a multicentre, randomised, placebo-controlled phase 3 trial[J]. Lancet, 2020,395(10236):1547-1557.

[76] Powles T, Park S H, Voog E, et al. Avelumab Maintenance Therapy for Advanced or Metastatic Urothelial Carcinoma[J]. N Engl J Med, 2020,383(13):1218-1230.

[77] Mittendorf E A, Zhang H, Barrios C H, et al. Neoadjuvant atezolizumab in combination with sequential nab-paclitaxel and anthracycline-based chemotherapy versus placebo and chemotherapy in patients with early-stage triple-negative breast cancer (IMpassion031): a randomised, double-blind, phase 3 trial[J]. Lancet, 2020,396(10257):1090-1100.

[78] Schmid P, Cortes J, Pusztai L, et al. Pembrolizumab for Early Triple-Negative Breast Cancer[J]. N Engl J Med, 2020,382(9):810-821.

[79] Huang J, Xu J, Chen Y, et al. Camrelizumab versus investigator's choice of chemotherapy as second-line therapy for advanced or metastatic oesophageal squamous cell carcinoma (ESCORT): a multicentre, randomised, open-label, phase 3 study[J]. Lancet Oncol, 2020,21(6):832-842.

[80] Kojima T, Shah M A, Muro K, et al. Randomized Phase III KEYNOTE-181 Study of Pembrolizumab Versus Chemotherapy in Advanced Esophageal Cancer[J]. J Clin Oncol, 2020,38(35):4138-4148.

[81] Even C, Wang H M, Li S H, et al. Phase II, Randomized Study of Spartalizumab (PDR001), an Anti-PD-1 Antibody, versus Chemotherapy in Patients with Recurrent/Metastatic Nasopharyngeal Cancer[J]. Clin Cancer Res, 2021,27(23):6413-6423.

[82] Sugawara S, Lee J S, Kang J H, et al. Nivolumab with carboplatin, paclitaxel, and bevacizumab for first-line treatment of advanced nonsquamous non-small-cell lung cancer[J]. Ann Oncol, 2021,32(9):1137-1147.

[83] Spigel D R, Vicente D, Ciuleanu T E, et al. Second-line nivolumab in relapsed small-cell lung cancer: CheckMate 331[J]. Ann Oncol, 2021,32(5):631-641.

[84] Owonikoko T K, Park K, Govindan R, et al. Nivolumab and Ipilimumab as Maintenance Therapy in Extensive-Disease Small-Cell Lung Cancer: CheckMate 451[J]. J Clin Oncol, 2021,39(12):1349-1359.

[85] Boyer M, Sendur M, Rodriguez-Abreu D, et al. Pembrolizumab Plus Ipilimumab or Placebo for Metastatic Non-Small-Cell Lung Cancer With PD-L1 Tumor Proportion Score >/= 50%: Randomized, Double-Blind Phase III KEYNOTE-598 Study[J]. J Clin Oncol, 2021,39(21):2327-2338.

[86] Lu S, Wang J, Yu Y, et al. Tislelizumab Plus Chemotherapy as First-Line Treatment for Locally Advanced or Metastatic Nonsquamous NSCLC (RATIONALE 304): A Randomized Phase 3 Trial[J]. J Thorac Oncol, 2021,16(9):1512-1522.

[87] Leighl N B, Laurie S A, Goss G D, et al. CCTG BR34: A Randomized Phase 2 Trial of Durvalumab and Tremelimumab With or Without Platinum-Based Chemotherapy in Patients With Metastatic NSCLC[J]. J Thorac Oncol, 2022,17(3):434-445.

[88] Gettinger S N, Redman M W, Bazhenova L, et al. Nivolumab Plus Ipilimumab vs Nivolumab for Previously Treated Patients With Stage IV Squamous Cell Lung Cancer: The Lung-MAP S1400I Phase 3 Randomized Clinical Trial[J]. JAMA Oncol, 2021,7(9):1368-1377.

[89] Ren S, Chen J, Xu X, et al. Camrelizumab Plus Carboplatin and Paclitaxel as First-Line Treatment for Advanced Squamous NSCLC (CameL-Sq): A Phase 3 Trial[J]. J Thorac Oncol, 2022,17(4):544-557.

[90] Zhou C, Wu L, Fan Y, et al. Sintilimab Plus Platinum and Gemcitabine as First-Line Treatment for Advanced or Metastatic Squamous NSCLC: Results From a Randomized, Double-Blind, Phase 3 Trial (ORIENT-12)[J]. J Thorac Oncol, 2021,16(9):1501-1511.

[91] Zhou C, Chen G, Huang Y, et al. Camrelizumab plus carboplatin and pemetrexed versus chemotherapy alone in chemotherapy-naive patients with advanced non-squamous non-small-cell lung cancer (CameL): a randomised, open-label, multicentre, phase 3 trial[J]. Lancet Respir Med, 2021,9(3):305-314.

[92] Wang J, Lu S, Yu X, et al. Tislelizumab Plus Chemotherapy vs Chemotherapy Alone as First-line Treatment for Advanced Squamous Non-Small-Cell Lung Cancer: A Phase 3 Randomized Clinical Trial[J]. JAMA Oncol, 2021,7(5):709-717.

[93] Peters S, Pujol J L, Dafni U, et al. Consolidation nivolumab and ipilimumab versus observation in limited-disease small-cell lung cancer after chemo-radiotherapy - results from the randomised phase II ETOP/IFCT 4-12 STIMULI trial[J]. Ann Oncol, 2022,33(1):67-79.

[94] Ren Z, Xu J, Bai Y, et al. Sintilimab plus a bevacizumab biosimilar (IBI305) versus sorafenib in unresectable hepatocellular carcinoma (ORIENT-32): a randomised, open-label, phase 2-3 study[J]. Lancet Oncol, 2021,22(7):977-990.

[95] Yau T, Park J W, Finn R S, et al. Nivolumab versus sorafenib in advanced hepatocellular carcinoma (CheckMate 459): a randomised, multicentre, open-label, phase 3 trial[J]. Lancet Oncol, 2022,23(1):77-90.

[96] Gogas H, Dreno B, Larkin J, et al. Cobimetinib plus atezolizumab in BRAF(V600) wild-type melanoma: primary results from the randomized phase III IMspire170 study[J]. Ann Oncol, 2021,32(3):384-394.

[97] Fennell D A, Ewings S, Ottensmeier C, et al. Nivolumab versus placebo in patients with relapsed malignant mesothelioma (CONFIRM): a multicentre, double-blind, randomised, phase 3 trial[J]. Lancet Oncol, 2021,22(11):1530-1540.

[98] Kuruvilla J, Ramchandren R, Santoro A, et al. Pembrolizumab versus brentuximab vedotin in relapsed or refractory classical Hodgkin lymphoma (KEYNOTE-204): an interim analysis of a multicentre, randomised, open-label, phase 3 study[J]. Lancet Oncol, 2021,22(4):512-524.

[99] Spigel D, Jotte R, Nemunaitis J, et al. Randomized Phase 2 Studies of Checkpoint Inhibitors Alone or in Combination With Pegilodecakin in Patients With Metastatic NSCLC (CYPRESS 1 and CYPRESS 2)[J]. J Thorac Oncol, 2021,16(2):327-333.

[100] Moore K N, Bookman M, Sehouli J, et al. Atezolizumab, Bevacizumab, and Chemotherapy for Newly Diagnosed Stage III or IV Ovarian Cancer: Placebo-Controlled Randomized Phase III Trial (IMagyn050/GOG 3015/ENGOT-OV39) [J]. J Clin Oncol, 2021,39(17): 1842-1855.

[101] Hamanishi J, Takeshima N, Katsumata N, et al. Nivolumab Versus Gemcitabine or Pegylated Liposomal Doxorubicin for Patients With Platinum-Resistant Ovarian Cancer: Open-Label, Randomized Trial in Japan (NINJA)[J]. J Clin Oncol, 2021,39(33):3671-3681.

[102] Pujade-Lauraine E, Fujiwara K, Ledermann J A, et al. Avelumab alone or in combination with chemotherapy versus chemotherapy alone in platinum-resistant or platinum-refractory ovarian cancer (JAVELIN Ovarian 200): an open-label, three-arm, randomised, phase 3 study[J]. Lancet Oncol, 2021,22(7):1034-1046.

[103] Monk B J, Colombo N, Oza A M, et al. Chemotherapy with or without avelumab followed by avelumab maintenance versus chemotherapy alone in patients with previously untreated epithelial ovarian cancer (JAVELIN Ovarian 100): an open-label, randomised, phase 3 trial[J]. Lancet Oncol, 2021,22(9):1275-1289.

[104] Bajorin D F, Witjes J A, Gschwend J E, et al. Adjuvant Nivolumab versus Placebo in Muscle-Invasive Urothelial Carcinoma[J]. N Engl J Med, 2021,384(22):2102-2114.

[105] Bellmunt J, Hussain M, Gschwend J E, et al. Adjuvant atezolizumab versus observation in muscle-invasive urothelial carcinoma (IMvigor010): a multicentre, open-label, randomised, phase 3 trial[J]. Lancet Oncol, 2021,22(4):525-537.

[106] Shah M A, Cunningham D, Metges J P, et al. Randomized, open-label, phase 2 study of andecaliximab plus nivolumab versus nivolumab alone in advanced gastric cancer identifies biomarkers associated with survival[J]. J Immunother Cancer, 2021,9(12).

[107] Powles T, Csoszi T, Ozguroglu M, et al. Pembrolizumab alone or combined with chemotherapy versus chemotherapy as first-line therapy for advanced urothelial carcinoma (KEYNOTE-361): a randomised, open-label, phase 3 trial[J]. Lancet Oncol, 2021,22(7):931-945.

[108] Miles D, Gligorov J, Andre F, et al. Primary results from IMpassion131, a double-blind, placebo-controlled, randomised phase III trial of first-line paclitaxel with or without atezolizumab for unresectable locally advanced/metastatic triple-negative breast cancer[J]. Ann Oncol, 2021,32(8):994-1004.

[109] Winer E P, Lipatov O, Im S A, et al. Pembrolizumab versus investigator-choice chemotherapy for metastatic triple-negative breast cancer (KEYNOTE-119): a randomised, open-label, phase 3 trial[J]. Lancet Oncol, 2021,22(4):499-511.

[110] Paz-Ares L, Ciuleanu T E, Cobo M, et al. First-line nivolumab plus ipilimumab combined with two cycles of chemotherapy in patients with non-small-cell lung cancer (CheckMate 9LA): an international, randomised, open-label, phase 3 trial[J]. Lancet Oncol, 2021,22(2):198-211.

[111] Felip E, Altorki N, Zhou C, et al. Adjuvant atezolizumab after adjuvant chemotherapy in resected stage IB-IIIA non-small-cell lung cancer (IMpower010): a randomised, multicentre, open-label, phase 3 trial[J]. Lancet, 2021,398(10308):1344-1357.

[112] Sezer A, Kilickap S, Gumus M, et al. Cemiplimab monotherapy for first-line treatment of advanced non-small-cell lung cancer with PD-L1 of at least 50%: a multicentre, open-label, global, phase 3, randomised, controlled trial[J]. Lancet, 2021,397(10274):592-604.

[113] Choueiri T K, Powles T, Burotto M, et al. Nivolumab plus Cabozantinib versus Sunitinib for Advanced Renal-Cell Carcinoma[J]. N Engl J Med, 2021,384(9):829-841.

[114] Choueiri T K, Tomczak P, Park S H, et al. Adjuvant Pembrolizumab after Nephrectomy in Renal-Cell Carcinoma[J]. N Engl J Med, 2021,385(8):683-694.

[115] Motzer R, Alekseev B, Rha S Y, et al. Lenvatinib plus Pembrolizumab or Everolimus for Advanced Renal Cell Carcinoma[J]. N Engl J Med, 2021,384(14):1289-1300.

[116] Sun J M, Shen L, Shah M A, et al. Pembrolizumab plus chemotherapy versus chemotherapy alone for first-line treatment of advanced oesophageal cancer (KEYNOTE-590): a randomised, placebo-controlled, phase 3 study[J]. Lancet, 2021,398(10302):759-771.

[117] Luo H, Lu J, Bai Y, et al. Effect of Camrelizumab vs Placebo Added to Chemotherapy on Survival and Progression-Free Survival in Patients With Advanced or Metastatic Esophageal Squamous Cell Carcinoma: The ESCORT-1st Randomized Clinical Trial[J]. JAMA, 2021,326(10):916-925.

[118] Kelly R J, Ajani J A, Kuzdzal J, et al. Adjuvant Nivolumab in Resected Esophageal or Gastroesophageal Junction Cancer[J]. N Engl J Med, 2021,384(13):1191-1203.

[119] Lee N Y, Ferris R L, Psyrri A, et al. Avelumab plus standard-of-care chemoradiotherapy versus chemoradiotherapy alone in patients with locally advanced squamous cell carcinoma of the head and neck: a randomised, double-blind, placebo-controlled, multicentre, phase 3 trial[J]. Lancet Oncol, 2021,22(4):450-462.

[120] Moehler M, Dvorkin M, Boku N, et al. Phase III Trial of Avelumab Maintenance After First-Line Induction Chemotherapy Versus Continuation of Chemotherapy in Patients With Gastric Cancers: Results From JAVELIN Gastric 100[J]. J Clin Oncol, 2021,39(9):966-977.

[121] Janjigian Y Y, Shitara K, Moehler M, et al. First-line nivolumab plus chemotherapy versus chemotherapy alone for advanced gastric, gastro-oesophageal junction, and oesophageal adenocarcinoma (CheckMate 649): a randomised, open-label, phase 3 trial[J]. Lancet, 2021,398(10294):27-40.

[122] Baas P, Scherpereel A, Nowak A K, et al. First-line nivolumab plus ipilimumab in unresectable malignant pleural mesothelioma (CheckMate 743): a multicentre, randomised, open-label, phase 3 trial[J]. Lancet, 2021,397(10272):375-386.

[123] Colombo N, Dubot C, Lorusso D, et al. Pembrolizumab for Persistent, Recurrent, or Metastatic Cervical Cancer[J]. N Engl J Med, 2021,385(20):1856-1867.
